# Comparing Nursing Student Competence in CPR before and after a Pedagogical Intervention

**DOI:** 10.1155/2020/7459084

**Published:** 2020-02-21

**Authors:** Siv Roel, Ida Torunn Bjørk

**Affiliations:** ^1^Department of Nursing and Health Science, University of South-Eastern Norway, Postboks 235, 3603 Kongsberg, Notodden, Norway; ^2^Department of Nursing Science, University of Oslo, Postboks 1130, Blindern, 0318 Oslo, Norway

## Abstract

Nursing students must be able to initiate and perform effective cardiopulmonary resuscitation (CPR) when they start their career in nursing. Studies show that students' competency in CPR is deficient, indicating that better training is necessary during nursing education. This study reports on the differences in nursing students' competence in CPR before and after a longitudinal pedagogical intervention across the curriculum. Changes in the curriculum were relocation and added testing of CPR skills, inclusion of a course in defibrillation, a knowledge test as stimuli before simulation, and more simulation practice with deteriorating patients. This was a comparative study between two cohorts of students in the bachelor in nursing education. We measured knowledge and compression performance in the students' final year of education. Students in cohort 2, who received the pedagogical intervention, had a significant higher total knowledge score than students in cohort 1. Students' mean depth and number of correct compressions was similar. Students in cohort 2 had a significantly higher mean rate of compressions, number of compressions per minute, and mean number of compressions with incorrect hand positions. Although the new curriculum afforded more hands-on practice of CPR, it was not enough to improve the students' performance to match the demands set out in national and international guidelines.

## 1. Introduction

Nursing students must be able to initiate and perform effective cardiopulmonary resuscitation (CPR) when they start their career in nursing [1, 2]. Often, the nurse arrives first at the scene of a cardiac arrest in the hospital [3, 4]. As it is the first few minutes with optimal CPR that are decisive for the patient's chances to survive the arrest, and for further quality of life, the nurse's competency in CPR is crucial. However, studies show that both nurses' and physicians' skill in CPR is deficient [5–7], indicating that better education in CPR and maintenance of skill is necessary. Educational institutions in nursing have a responsibility to qualify nursing students in CPR through well-developed basic life support programs. To our knowledge there are few studies that explore how different curricula in CPR affect students' learning outcomes. A pedagogical intervention across three years of bachelor nursing education was developed to increase nursing students' learning outcomes in basic life support including CRP. In the present study, we compare nursing students' competence in CPR before and after the pedagogical intervention.

Due to the advancement of simulation technologies, several studies have explored the effect on nursing students' CPR skills when the teaching methods incorporate simulators with different levels of fidelity. Students training on a high fidelity simulator achieved significantly higher scores on knowledge and skill than students training on low fidelity simulators [8]. When high fidelity simulators were used as an enhancement in experimental groups, scores on both knowledge [9, 10] and confidence [10] were significantly higher, and the students' adherence to basic life support guidelines [9] was significantly better, compared to scores in the control groups. In general, learning with high fidelity simulators does not always yield better results than learning with low fidelity simulators. Both the type of skill and the learner's educational level will influence the results [11]. Confidence is a self-reported measure that may be of limited value [12]. For example, Liaw et al. [13] found no correlation between students' self-reported confidence and skills performance. Roh and Kim [14] introduced self-directed computer-based simulation training as an enhancement to one group of students after the traditional instructor-led simulation in both control and experimental group. No significant differences were found between the groups' scores on either team performance, self-efficacy, postcode stress, or satisfaction with simulation.

Aspects of the instructor in CPR teaching have been explored in several studies. Kim and Roh [3] stated that CPR education was vulnerable to the instructors' teaching quality and to the design of CPR education. They found a general mismatch between what the instructors claimed was important in CPR education and what they actually did. In other studies, findings have shown that self-directed learning was better than instructor-led learning [15], training with voice advisory manikins (VAM) was better than instructor-led training [4], and the debriefing after CPR was better if it was instructor-led than peer-led [16].

Retention of nursing students' CPR skills and knowledge has been tested in several studies, approximately 3 months after initial training. Mostly, both knowledge and skills deteriorate in students over time [8, 9, 17], although Partiprajak and Thongpo [18] reported that a group of 30 students included in their study on an average maintained their skill performance scores after 3 months. To test for improvement in skills retention, Oermann et al. [19] designed a study where students in the experimental group repeated CPR practice 6 minutes every month for one year on a voice advisory manikin. They concluded that only 6 minutes training resulted in maintenance of both compression rate and depth in the experimental group, while students in the control group maintained their compression rate. The aim of the present study was to compare nursing students' knowledge and skill in CPR before and after a pedagogical intervention. We hypothesized that students in cohort 2 would demonstrate more knowledge and skill in CPR after following the new CPR education program. We developed the following research question:

Is there a difference in nursing student competence in CPR before and after a pedagogical intervention, measured by a knowledge test and by compression parameters, available during CPR?

## 2. Material and Methods

### 2.1. Design

This is a comparative study [20] of skill and knowledge acquisition in CPR between two cohorts of students in the bachelor in nursing education. Cohort 1 followed the existing study program, while cohort 2 experienced CPR education based on a new pedagogical design. Data was collected from cohort 1 in spring 2014 and from cohort 2 in autumn 2014.

### 2.2. Participants and Setting

The study was conducted at a university college in the southeastern part of Norway. A total of 145 students in their last year of a 3-year bachelor in nursing program were invited, and 142 students agreed to participate (98%). Cohort 1 comprised 60 students, 52 women, and 8 men from the part-time program. Average age of the students at completion of the program was 28 years (range 21–47). Cohort 2 comprised 82 students, 75 women, and 7 men from the full-time program. Average age at the completion of the program was 25.5 (range 21–54). During training, students in both cohorts were divided into groups of 6–10.

### 2.3. Description of the CPR Education Program

CPR was embedded in the basic life support (BLS) education program during the 3-year bachelor in nursing education. In Table 1, we present an overview of the program for the two cohorts that show the changes in the curriculum across three years. These changes were based on the lacking competence that graduating nursing students exhibited during the third year emergency exercise in our university college. The changes were also based on findings from the research literature highlighting the need for structured pedagogical programs especially including more practical training of skills [9, 19, 21].

First year: education in basic life support, including CPR, was similar in both cohorts and included five lectures: basic first aid (two lectures), first aid with children (two lectures), and foreign-body airway obstruction (FBAO) (one lecture). Students continued with three hours of training in the simulation center with an instructor. Training time in the simulation center was equally divided between CPR on Little Anne® Torso (Laerdal Medical, Norway) and techniques to remove FBAO simulated with peer students. Students in cohort 2 were granted a three-month license period to practice CPR on Resusci® Anne Skills Station (Laerdal Medical, Norway). The skills station was connected to a PC with software that provided feedback on compression and ventilation. When students satisfied the parameters of the European Resuscitation Council Guidelines [22], the test was approved.

Second year: both cohorts participated in instructor-led simulation scenarios where one scenario included CPR on a patient that developed a cardiac arrest. Both cohorts practiced on Resusci® Anne SkillReporter™ (Laerdal Medical, Norway). In addition, cohort 2 completed a knowledge pretest as stimuli for learning (the test included 3 questions on CPR) one week before the simulation and had a longer debriefing session. Cohort 2 also participated in a defibrillation course (DCPR) consisting of an e-learning program and three hours of instructor-led training in CPR on Little Anne® Torso (Laerdal Medical, Norway). The defibrillator (AED) Trainer FR2 Norwegian version (Laerdal Medical, Norway) was used to practice defibrillation. Students had to practice until the instructor could certify their skills.

Third year: both cohorts participated in a nonhospital accident and emergency simulation that focused on nurses' special responsibilities in accidents and emergencies. Students rotated through four acute scenarios lasting approximately 60 min each, car accident, hypoglycemia and stroke, triage, and CPR on Resusci® Anne SkillReporter™ (Laerdal Medical, Norway). Students in both cohorts had the opportunity to try out the defibrillator AED Trainer FR2 Norwegian version (Laerdal Medical, Norway). Cohort 1 was tested in CPR earlier in the third year.

### 2.4. Development of the Questionnaire

A knowledge test was developed based on the content in the Norwegian national course in CPR [23], including use of a defibrillator AED Trainer FR2 (Laerdal Medical, Norway). The course was based on the European Resuscitation Council Guidelines [22]. The test included eight questions covering the following content: (1) heart attack, (2) unexpected cardiac arrest, (3) vital signs, (4) ventilation, (5) AED's function on the heart, (6) when and who will use the AED, (7) placement of the AED electrodes, and (8) technical function of the AED. Each question had four possible answers. The students had to mark off two correct answers on each question. Both answers had to be correct for the student to get 1 point per question.

### 2.5. Data Collection

Compression data were collected in both cohorts in the CPR scenario during the emergency exercise in the students' third year of education (Table 1). Prints of the following parameters were collected for comparison from all students: compression depth (depthc), number of compression per minute (numbercm), compression rate (ratec), correct compression (correctc), and compression with incorrect hand position (inadequate). Total number of compressions was not included because all students were tested for five minutes. Only one student compressed too deeply so that parameter was not included in the study. Students were tested on Resusci® Anne SkillReporter (Laerdal Medical, Norway). Data on ventilation were not included in the study as the ventilation readings from the manikin were not correct. The knowledge test was given directly after the CPR test as a paper and pencil test.

### 2.6. Ethical Considerations

The study received institutional approval from the dean and was reviewed and approved by the Norwegian Centre for Research Data. Students were informed in class and on the internal learning platform. All participating students signed an informed consent.

### 2.7. Analysis of Data

The SPSS version 23 was applied to examine the data. Compression data from the Resusci® Anne SkillReporter and knowledge scores was analyzed with descriptive statistics. The independent *t*-test was applied to assess the presence of any statistical significant differences between the two cohorts. The significance threshold was set at 0.05.

## 3. Results

Student scores on the knowledge test are presented in Table 2.

Cohort 2 had a significantly higher total knowledge score than cohort 1 (Table 2). Table 2 also shows that cohort 2 had higher scores on six of the eight questions. In four of these questions, the difference in scores was significant in favor of students in cohort 2. Three of these concerned function and use of the AED defibrillator; the fourth concerned the ability to decide about performing CPR based on different vital signs.

**Figure 1:**
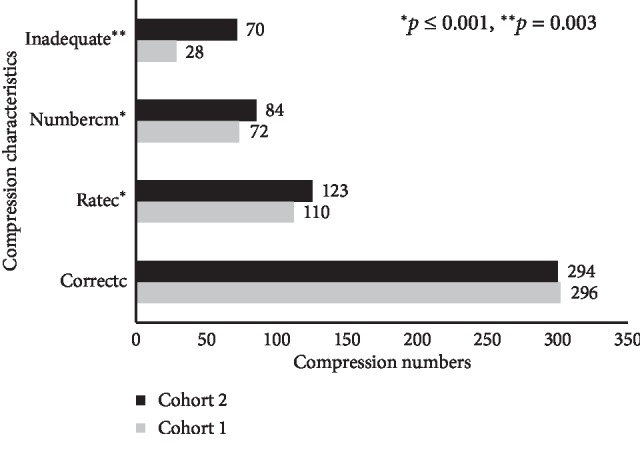
Comparison of compression parameters of the CPR test between the two cohorts.

The analysis of compression data showed that the students' mean depth of compression as measured in millimeters (mm) was quite similar; 55 mm and 54.4 mm in cohort 1 and 2, respectively. A comparison of the students' skill in the other compression parameters is presented in Figure 1.

Figure 1 shows that students had a similar mean number of correct compressions (correctc). Students in cohort 2 had a significantly higher mean rate of compressions (ratec) and number of compressions per minute (numbercm) and also a significantly higher mean number of compressions with incorrect hand positions (inadequate).

## 4. Discussion

The purpose of this study was to compare nursing students' competence in CPR in the form of cognitive knowledge and skills in compression before and after a longitudinal pedagogical intervention. Changes in the curriculum were firstly an increase and relocation in testing of CPR skills, increase from one to two tests, and relocation from 3^rd^ year to 1^st^ and 2^nd^ year. Secondly, a course in defibrillation was added. Thirdly, students in cohort 2 conducted a knowledge test as stimuli before simulation, and lastly, they had more simulation practice with deteriorating patients. In the following, the possible impact of these curriculum changes on the students' results will be discussed.

### 4.1. Students' Knowledge Scores

Students in cohort 2 had significantly higher overall knowledge scores than students in cohort 1. In general, a majority of studies show increased knowledge in students after attending simulation of varied fidelity [24, 25]. More recent studies corroborate these findings related to CPR knowledge [15, 18]. The increase in knowledge scores in cohort 2 in the present study are not related to differences in the type of simulation as all students used the same manikins from Laerdal. However, we suggest that the increase in knowledge scores is related to other aspects of the pedagogical intervention, a stimulus test, the course in defibrillation, and more simulated experiences with deteriorating patients. The stimulus test included three questions on CPR. A stimulus test intends to trigger students to brush up on lacking knowledge before simulation and was presented as a learning incentive in FIRST2ACT, a theory-based simulation model [26]. A stimulus test can uncover what the student knows and does not know, stimulate the student to check knowledge before simulation, and also function as a trigger to remind the student about important knowledge during the actual simulation [27].

Significant higher scores on knowledge was evident among students in cohort 2 related to three of four questions on defibrillation. It is natural to relate this to the fact that students in cohort 2 had a special course on defibrillation in their second year, while students in cohort 1 had the common lectures on basic first aid including theory on defibrillation and an offer to try defibrillation during the emergency exercises. An interesting aspect of the significant scores among students in cohort 2 is that students had the course on defibrillation one year before the tests in the present study. Retention of both knowledge and skill in CPR is a contentious issue among practitioners as well as students because scores on knowledge invariably are reduced when students are tested at a later stage [9, 15, 18]. Since the scores in the present study were actually quite high (means between 0.65 and 0.91 on questions 5-7 in Table 2), this indicates that students retained much of the knowledge acquired one year earlier, about use of the AED.

Students in cohort 2 also had significant higher scores on the question about deciding about CPR based on knowledge about vital signs. We can only speculate with regard to this finding that the increased amount of scenario practice with deteriorating patients may have influenced students' knowledge positively. Although only one scenario concerned a patient with cardiac arrest, all the other scenarios stimulated the students to check on patients' vital signs and to deliberate on the relationship between deterioration in vital signs and the patient's health problem.

### 4.2. Students' Skill in Compression

Characteristics of the two student cohorts' compressions were both similar and significantly different. Interestingly, the mean depth of compressions was well within parameters set by the European Resuscitation Council Guidelines [22] and American Heart Association [28] in both cohorts. High quality CPR skills must provide chest compressions of adequate depth [29], but former research shows that this is one objective that many nursing students struggle to fulfill. In the studies by Roh and Lim [30] and Roh and Issenberg [29], 58% and 93% of the students had insufficient depth of chest compressions, respectively. Oermann et al. [21] reported from a large study in the USA that students' depth of compression was between approximately 41-42 mm over a 12 month study with repeated practice. These numbers satisfied the AHA guidelines from 2005 [31], but only 11% of the students compressed to a level of 51 mm that would have satisfied the guidelines of 2010 [28]. A recent study from Thailand shows similar findings to ours; 87% of the students compressed sufficiently in a post-test after a 2-hour BLS course. A retest after 3 months showed even better results as 96.7% of the students' compressions were between 50-–60 mm. This was however a study with only 30 participating students [18].

The number of correct compressions was similar in the two cohorts while mean inadequate hand positioning was significantly higher among students in cohort 2 than in cohort 1. Only a few studies report on hand positioning, mostly as quite correct [18, 21]. Correct hand positioning will ideally secure more correct depth of the compressions, although in the present study all students performed acceptable and similar depth of compressions despite more incorrect hand positioning among students in cohort 2. This is probably related to the fact that students in cohort 2 also had a significantly higher rate and number of compressions per minute that probably compensated for inadequate hand positioning. Guidelines relevant for the student groups in this study advice a mean compression rate of 100–120 to secure enough actual compressions per minute in combination with ventilations [22, 28]; cohort 1 was within this range while cohort 2 was slightly above. Other studies show that students struggle to keep the rate of compressions within the appropriate range [18, 29]. The small differences detected between the two cohorts' compression scores indicate that the increase in practice of CPR in cohort 2 was not enough to improve the students' performance. Support for more and repeated practice of CPR is found in Oermann et al. [19] study, where students practicing CPR for six minutes a month for one year retained and improved their compression skills.

In light of our findings, there is still a need for a continued focus on CPR education for nursing students. However, the need for continued training in CPR is evidently also an issue after students finish their education, as studies show deteriorating competence in CPR among professional nurses as well [5–7]. It is a paradox that there seems to be a greater focus on the public's competence in CPR than the competence among health care personnel. This indicates the need for continued efforts to provide structured programs for CPR training in all clinical settings.

### 4.3. Strengths and Limitations

To our knowledge, this is the first study that compares the effect of a longitudinal curriculum change in basic life support including CPR on students' knowledge and performance skills. The sample included two cohorts of students but was limited to students at one university college. The two cohorts participated in a full-time and a part-time program. This may have influenced the results. However, the students followed the same curriculum and used the same time for each course. Another limitation is related to the difficulties in extracting correct data on students' ventilation of the manikins used in the study.

## 5. Conclusion

The present study reports on a comparison of students' knowledge and performance of CPR before and after a major change in the curriculum of basic life support including CPR. Students in cohort 2 followed a three-year curriculum with more practice and testing of CPR and more knowledge input. The findings show that changes in the curriculum positively influenced the knowledge scores of students in cohort 2. Both cohorts fulfilled the guideline claims of compression depth, but varied more concerning hand positioning and rate of compressions. Although the new curriculum afforded more hands-on practice of CPR, it was not enough to improve the students' performance to match the demands set out in national and international guidelines.

## Figures and Tables

**Table 1:** Structure of basic life support education including CPR.

	Cohort 1	Cohort 2
First year	Basic life support including CPR	Basic life support including CPR
		Self-organized practice with test in CPR (voice instruction)

Second year	3 scenario simulations with deteriorating patients—one scenario including CPR	6 scenario simulations with deteriorating patients—one scenario including CPR
		Knowledge test as stimuli before simulation
		Course and certification of skill in CPR with automated external defibrillation (DCPR)

Third year	Test in CPR (voice instruction)	Emergency exercise—4 scenario simulations of acute situations—one including CPR
	Emergency exercise—4 scenario simulations of acute situations—one including CPR	

**Table 2:** Scores on the knowledge test (range 1–8).

	Cohort 1, *n* = 60	Cohort 2, *n* = 82	*p* values
	Mean ± SD	Mean ± SD	
Total knowledge score	4.75 ± 1.67	6.06 ± 1.99	≤0.001
Q1: heart attack	0.80 ± 0.40	0.87 ± 0.34	ns
Q2: unexpected cardiac arrest	0.53 ± 0.50	0.48 ± 0.50	ns
Q3: vital signs	0.20 ± 0.40	0.72 ± 0.45	≤0.001
Q4: ventilation	0.93 ± 0.25	0.88 ± 0.33	ns
Q5: AED's function on the heart	0.53 ± 0.50	0.80 ± 0.40	= 0.001
Q6: when and who should use the AED	0.40 ± 0.49	0.65 ± 0.48	= 0.004
Q7: placement of the electrodes	0.73 ± 0.45	0.91 ± 0.28	= 0.007
Q8: technical function of AED	0.62 ± 0.49	0.76 ± 0.43	ns

## Data Availability

The underlying data will be available through the USN Research Data Archive after acceptance of the article.
